# Supplementary material for: Be positive: customized reference databases and new, local barcodes balance false taxonomic assignments in metabarcoding studies
Source: PeerJ. 2023 Jan 9;11:e14616. doi: 10.7717/peerj.14616 (PMC9835706; doi:10.7717/peerj.14616)
Supplement: Supplemental Information 5 [file peerj-11-14616-s005.html]

 Data\_S5-not-to-upload


# S5 Supplementary Information

**Be positive: customised reference databases and new, local barcodes balance false taxonomic assignments in metabarcoding studies**

*List of commands to produce customized reference databases and for taxonomic assignments of metabarcoding data.*

## Tools used in this workflow

- **COInr DB** https://zenodo.org/record/6555985, (Meglécz, 2022a, 2022b)
- **mkCOInr** v-0.2.0 https://github.com/meglecz/mkCOInr, (Meglécz, 2022b, 2022c)
- **VTAM** v-0.2.0 https://github.com/aitgon/vtam (González *et al.*, 2020)
- **RDP classifier** v-2.13 https://sourceforge.net/p/rdp-classifier/news/2020/07/rdp-classifier-213-july-2020-release-note/, (Wang *et al.*, 2007)
- **QIIME2** v-Core-2022.2 https://docs.qiime2.org/2022.8/install/, (Bolyen *et al.*, 2019)

# 1. Select sequences from COInr

## 1.1 Download COInr DB

Downloaded from www.zenodo.org/record/6555985.
This DB is ready to use as it is for formatting and for selecting a desired target region. It comes with an associated taxonomy .tsv file.

```
```
mkdir -p ~/DB_comp
```


```
cd ~/DB_comp
```


```
wget https://zenodo.org/record/6555985/files/COInr_2022_05_06.tar.gz
```


```
tar -zxvf COInr_2022_05_06.tar.gz
```


```
mv COInr_2022_05_06 COInr
```


```
rm COInr_2022_05_06.tar.gz
```
```

## 1.2 COInr-WO-Insecta

Remove insect sequences from COInr

```
```
xxxxxxxxxx
```


```
perl ~/mkCOInr/scripts/select_taxa.pl \
```


```
  -taxon_list metafiles/taxon_list_insecta.txt \
```


```
  -tsv COInr/COInr.tsv \
```


```
  -taxonomy COInr/taxonomy.tsv \
```


```
  -outdir COInr_WO_Insecta/ \
```


```
  -out COInr_WO_Insecta.tsv \
```


```
  -negative_list 1
```
```

*metafiles/taxon\_list\_insecta.txt*:

```
```
xxxxxxxxxx
```


```
taxon_name
```


```
Insecta
```
```

## 1.3 COInr-Med

Derived from COInr-WO-Insecta and refined for Mediterranean marine Families gathered from OBIS\*

```
```
xxxxxxxxxx
```


```
perl ~/mkCOInr/scripts/select_taxa.pl \
```


```
  -taxon_list metafiles/Data_S4.tsv \
```


```
  -tsv COInr_WO_Insecta/COInr_WO_Insecta.tsv \
```


```
  -taxonomy COInr/taxonomy.tsv \
```


```
  -outdir COInr_Med/ \
```


```
  -out COInr_Med.tsv \
```


```
  -negative_list 0
```
```

*Data\_S4.tsv* is the list of taxonomic families present in the Mediterranean Sea.

## 1.4 COInr-Med+

Add new barcodes to COInr-Med

### 1.4.1 Suggest one or more lineages for each taxon name based on the existing lineages in taxonomy.tsv

```
```
xxxxxxxxxx
```


```
  perl ~/mkCOInr/scripts/format_custom.pl \
```


```
  -custom metafiles/Data_S2_barcodes.tsv \
```


```
  -taxonomy COInr/taxonomy.tsv \
```


```
  -outdir COInr_Med_plus/format_custom
```
```

*Data\_S2\_barcodes.tsv* is a tab separated file with seqID, taxon, sequence as columns. It can be created from Data\_S2.tsv by selecting the appropriate columns.

```
```
xxxxxxxxxx
```


```
seqID   taxon      sequence
```


```
Seq1 Orbinia sertulata      ATCAGTAGATATAGCAATC...
```


```
Seq10    Achelia langi      TTTATCATCGAGATTGGC...
```
```

The output is a lineage file *COInr\_Med\_plus/format\_custom/custom\_lineages.tsv* and a sequence file *COInr\_Med\_plus/format\_custom/custom\_sequences.tsv*.

Revise the output lineage file to complete lineages if taxon name is new to taxonomy.tsv and choose between homonyms if necessary.

*custom\_lineages\_verified.tsv*

```
```
xxxxxxxxxx
```


```
phylum  class   order   family  subfamily       genus   species seqIDs
```


```
Cnidaria    Anthozoa    Pennatulacea    Funiculinidae       Funiculina  Funiculina quadrangularis   Seq103;Seq130
```


```
...
```
```

### 1.4.2 Find taxID for each taxon in the lineage file

```
```
xxxxxxxxxx
```


```
perl ~/mkCOInr/scripts/add_taxids.pl \
```


```
  -lineages metafiles/custom_lineages_verified.tsv \
```


```
  -sequences COInr_Med_plus/format_custom/custom_sequences.tsv \
```


```
  -outdir COInr_Med_plus/add_taxids \
```


```
  -taxonomy COInr/taxonomy.tsv
```
```

This command will update the taxonomy.tsv file by adding new taxIDs. Remember to use the generated *COInr\_Med\_plus/add\_taxids/taxonomy\_updated.tsv* file for further taxonomic assignation steps.
It will also produce *COInr\_Med\_plus/add\_taxids/sequences\_with\_taxIDs.tsv* used in the next step.

### 1.4.3 Dereplicate custom sequences

```
```
xxxxxxxxxx
```


```
perl ~/mkCOInr/scripts/dereplicate.pl \
```


```
  -tsv COInr_Med_plus/add_taxids/sequences_with_taxIDs.tsv \
```


```
  -outdir COInr_Med_plus/dereplicate \
```


```
  -out custom_dereplicated_sequences.tsv
```
```

### 1.4.4 Pool and dereplicate COInr\_Med DB + custom sequences

```
```
xxxxxxxxxx
```


```
perl ~/mkCOInr/scripts/pool_and_dereplicate.pl \
```


```
  -tsv1 COInr_Med/COInr_Med.tsv \
```


```
  -tsv2 COInr_Med_plus/dereplicate/custom_dereplicated_sequences.tsv \
```


```
  -outdir COInr_Med_plus \
```


```
  -out COInr_Med_plus.tsv
```
```

Move the updated taxonomy file to the same folder as the COInr\_Med\_plus.tsv.

```
```
xxxxxxxxxx
```


```
mv COInr_Med_plus/add_taxids/taxonomy_updated.tsv COInr_Med_plus
```
```

# 2. Select the Leray region for each DB

Select sequences that cover at least 80% of the region amplified by metabarcoding primer pairs and trim sequences to this region.

## 2.1 COInr

```
```
xxxxxxxxxx
```


```
perl ~/mkCOInr/scripts/select_region.pl \
```


```
  -tsv COInr/COInr.tsv \
```


```
  -outdir leray/COInr \
```


```
  -e_pcr 1 \
```


```
  -fw GGNTGAACNGTNTAYCCNCC \
```


```
  -rv TAWACTTCDGGRTGNCCRAARAAYCA \
```


```
  -trim_error 0.3 \
```


```
  -min_amplicon_length 280 \
```


```
  -max_amplicon_length 345 \
```


```
  -min_overlap 20 \
```


```
  -tcov 0.8 \
```


```
  -identity 0.7
```
```

## 2.2 COInr-WO-Insecta

```
```
xxxxxxxxxx
```


```
perl ~/mkCOInr/scripts/select_region.pl \
```


```
  -tsv COInr_WO_Insecta/COInr_WO_Insecta.tsv \
```


```
  -outdir leray/COInr_WO_Insecta \
```


```
  -e_pcr 1 \
```


```
  -fw GGNTGAACNGTNTAYCCNCC \
```


```
  -rv TAWACTTCDGGRTGNCCRAARAAYCA \
```


```
  -trim_error 0.3 \
```


```
  -min_amplicon_length 280 \
```


```
  -max_amplicon_length 345 \
```


```
  -min_overlap 20 \
```


```
  -tcov 0.8 \
```


```
  -identity 0.7
```
```

## 2.3 COInr-Med

```
```
xxxxxxxxxx
```


```
perl ~/mkCOInr/scripts/select_region.pl \
```


```
  -tsv COInr_Med/COInr_Med.tsv \
```


```
  -outdir leray/COInr_Med \
```


```
  -e_pcr 1 \
```


```
  -fw GGNTGAACNGTNTAYCCNCC \
```


```
  -rv TAWACTTCDGGRTGNCCRAARAAYCA \
```


```
  -trim_error 0.3 \
```


```
  -min_amplicon_length 280 \
```


```
  -max_amplicon_length 345 \
```


```
  -min_overlap 20 \
```


```
  -tcov 0.8 \
```


```
  -identity 0.7
```
```

## 2.4 COInr-Med+

```
```
xxxxxxxxxx
```


```
perl ~/mkCOInr/scripts/select_region.pl \
```


```
  -tsv COInr_Med_plus/COInr_Med_plus.tsv \
```


```
  -outdir leray/COInr_Med_plus \
```


```
  -e_pcr 1 \
```


```
  -fw GGNTGAACNGTNTAYCCNCC \
```


```
  -rv TAWACTTCDGGRTGNCCRAARAAYCA \
```


```
  -trim_error 0.3 \
```


```
  -min_amplicon_length 280 \
```


```
  -max_amplicon_length 345 \
```


```
  -min_overlap 20 \
```


```
  -tcov 0.8 \
```


```
  -identity 0.7
```
```

# 3. Format the four databases for each taxassing tools

## 3.1 VTAM format

### 3.1.1 COInr

```
```
xxxxxxxxxx
```


```
perl ~/mkCOInr/scripts/format_db.pl \
```


```
  -tsv leray/COInr/trimmed.tsv \
```


```
  -taxonomy COInr/taxonomy.tsv \
```


```
  -outfmt vtam \
```


```
  -outdir leray/COInr/vtam \
```


```
  -out COInr_vtam
```
```

### 3.1.2 COInr-WO-Insecta

```
```
xxxxxxxxxx
```


```
perl ~/mkCOInr/scripts/format_db.pl \
```


```
  -tsv leray/COInr_WO_Insecta/trimmed.tsv \
```


```
  -taxonomy COInr/taxonomy.tsv \
```


```
  -outfmt vtam \
```


```
  -outdir leray/COInr_WO_Insecta/vtam \
```


```
  -out COInr_WO_Insecta_vtam
```
```

### 3.1.3 COInr-Med

```
```
xxxxxxxxxx
```


```
perl ~/mkCOInr/scripts/format_db.pl \
```


```
  -tsv leray/COInr_Med/trimmed.tsv \
```


```
  -taxonomy COInr/taxonomy.tsv \
```


```
  -outfmt vtam \
```


```
  -outdir leray/COInr_Med/vtam \
```


```
  -out COInr_Med_vtam
```
```

### 3.1.4 COInr-Med+

```
```
xxxxxxxxxx
```


```
perl ~/mkCOInr/scripts/format_db.pl \
```


```
  -tsv leray/COInr_Med_plus/trimmed.tsv \
```


```
  -taxonomy COInr_Med_plus/taxonomy_updated.tsv \
```


```
  -outfmt vtam \
```


```
  -outdir leray/COInr_Med_plus/vtam \
```


```
  -out COInr_Med_plus_vtam
```
```

## 3.2 RDP format

### 3.2.1 COInr

```
```
xxxxxxxxxx
```


```
perl ~/mkCOInr/scripts/format_db.pl \
```


```
  -tsv leray/COInr/trimmed.tsv \
```


```
  -taxonomy COInr/taxonomy.tsv \
```


```
  -outfmt rdp \
```


```
  -outdir leray/COInr/rdp \
```


```
  -out COInr_rdp
```
```

### 3.2.2 COInr-WO-Insecta

```
```
xxxxxxxxxx
```


```
perl ~/mkCOInr/scripts/format_db.pl \
```


```
  -tsv leray/COInr_WO_Insecta/trimmed.tsv \
```


```
  -taxonomy COInr/taxonomy.tsv \
```


```
  -outfmt rdp \
```


```
  -outdir leray/COInr_WO_Insecta/rdp \
```


```
  -out COInr_WO_Insecta_rdp
```
```

### 3.2.3 COInr-Med

```
```
xxxxxxxxxx
```


```
perl ~/mkCOInr/scripts/format_db.pl \
```


```
  -tsv leray/COInr_Med/trimmed.tsv \
```


```
  -taxonomy COInr/taxonomy.tsv \
```


```
  -outfmt rdp \
```


```
  -outdir leray/COInr_Med/rdp \
```


```
  -out COInr_Med_rdp
```
```

### 3.2.4 COInr-Med+

```
```
xxxxxxxxxx
```


```
perl ~/mkCOInr/scripts/format_db.pl \
```


```
  -tsv leray/COInr_Med_plus/trimmed.tsv \
```


```
  -taxonomy COInr_Med_plus/taxonomy_updated.tsv \
```


```
  -outfmt rdp \
```


```
  -outdir leray/COInr_Med_plus/rdp \
```


```
  -out COInr_Med_plus_rdp
```
```

## 3.3 QIIME format

### 3.3.1 COInr

```
```
xxxxxxxxxx
```


```
perl ~/mkCOInr/scripts/format_db.pl \
```


```
  -tsv leray/COInr/trimmed.tsv \
```


```
  -taxonomy COInr/taxonomy.tsv \
```


```
  -outfmt qiime \
```


```
  -outdir leray/COInr/qiime \
```


```
  -out COInr_qiime
```
```

### 3.3.2 COInr-WO-Insecta

```
```
xxxxxxxxxx
```


```
perl ~/mkCOInr/scripts/format_db.pl \
```


```
  -tsv leray/COInr_WO_Insecta/trimmed.tsv \
```


```
  -taxonomy COInr/taxonomy.tsv \
```


```
  -outfmt qiime \
```


```
  -outdir leray/COInr_WO_Insecta/qiime \
```


```
  -out COInr_WO_Insecta_qiime
```
```

### 3.3.3 COInr-Med

```
```
xxxxxxxxxx
```


```
perl ~/mkCOInr/scripts/format_db.pl \
```


```
  -tsv leray/COInr_Med/trimmed.tsv \
```


```
  -taxonomy COInr/taxonomy.tsv \
```


```
  -outfmt qiime \
```


```
  -outdir leray/COInr_Med/qiime \
```


```
  -out COInr_Med_qiime
```
```

### 3.3.4 COInr-Med+

```
```
xxxxxxxxxx
```


```
perl ~/mkCOInr/scripts/format_db.pl \
```


```
  -tsv leray/COInr_Med_plus/trimmed.tsv \
```


```
  -taxonomy COInr_Med_plus/taxonomy_updated.tsv \
```


```
  -outfmt qiime \
```


```
  -outdir leray/COInr_Med_plus/qiime \
```


```
  -out COInr_Med_plus_qiime
```
```

# 4. Taxonomic assignment

## 4.1 VTAM taxassign

Create output directory

```
```
xxxxxxxxxx
```


```
mkdir -p taxassign/vtam
```
```

### 4.1.1 COInr

```
```
xxxxxxxxxx
```


```
vtam taxassign \
```


```
  --mode reset \
```


```
  --db metafiles/db.sqlite \
```


```
  --asvtable metafiles/Data_S3.tsv \
```


```
  --output taxassign/vtam/COInr_vtam_taxassign.tsv \
```


```
  --taxonomy leray/COInr/vtam/COInr_vtam_taxonomy.tsv \
```


```
  --blastdbdir leray/COInr/vtam/ \
```


```
  --blastdbname COInr_vtam \
```


```
  -v
```
```

### 4.1.2 COInr-WO-Insecta

```
```
xxxxxxxxxx
```


```
vtam taxassign \
```


```
  --mode reset \
```


```
  --db metafiles/db.sqlite \
```


```
  --asvtable metafiles/Data_S3.tsv \
```


```
  --output taxassign/vtam/COInr_WO_Insecta_vtam_taxassign.tsv \
```


```
  --taxonomy leray/COInr_WO_Insecta/vtam/COInr_WO_Insecta_vtam_taxonomy.tsv \
```


```
  --blastdbdir leray/COInr_WO_Insecta/vtam/ \
```


```
  --blastdbname COInr_WO_Insecta_vtam \
```


```
  -v
```
```

### 4.1.3 COInr-Med

```
```
xxxxxxxxxx
```


```
vtam taxassign \
```


```
  --mode reset \
```


```
  --db metafiles/db.sqlite \
```


```
  --asvtable metafiles/Data_S3.tsv \
```


```
  --output taxassign/vtam/COInr_Med_vtam_taxassign.tsv \
```


```
  --taxonomy leray/COInr_Med/vtam/COInr_Med_vtam_taxonomy.tsv \
```


```
  --blastdbdir leray/COInr_Med/vtam/ \
```


```
  --blastdbname COInr_Med_vtam \
```


```
  -v
```
```

### 4.1.4 COInr-Med+

```
```
xxxxxxxxxx
```


```
vtam taxassign \
```


```
  --mode reset \
```


```
  --db metafiles/db.sqlite \
```


```
  --asvtable metafiles/Data_S3.tsv \
```


```
  --output taxassign/vtam/COInr_Med_plus_vtam_taxassign.tsv \
```


```
  --taxonomy leray/COInr_Med_plus/vtam/COInr_Med_plus_vtam_taxonomy.tsv \
```


```
  --blastdbdir leray/COInr_Med_plus/vtam/ \
```


```
  --blastdbname COInr_Med_plus_vtam \
```


```
  -v
```
```

## 4.2 RDP classifier

### 4.2.1 RDP training

The "Xmx216g" command has to be adjusted according to your available RAM (e.g., 216 = 216GB).
Do not use all the available RAM of your machine, it will freeze.

Create output directories

```
```
xxxxxxxxxx
```


```
mkdir -p leray/COInr/rdp/trained
```


```
mkdir -p leray/COInr_WO_Insecta/rdp/trained
```


```
mkdir -p leray/COInr_Med/rdp/trained
```


```
mkdir -p leray/COInr_Med_plus/rdp/trained
```
```

#### COInr

```
```
xxxxxxxxxx
```


```
java \
```


```
  -Xmx216g \
```


```
  -jar rdp_classifier_2.13/rdp_classifier_2.13/dist/classifier.jar \
```


```
  train \
```


```
  -o leray/COInr/rdp/trained/ \
```


```
  -s leray/COInr/rdp/COInr_rdp_trainseq.fasta \
```


```
  -t leray/COInr/rdp/COInr_rdp_taxon.txt
```
```

#### COInr-WO-Insecta

```
```
xxxxxxxxxx
```


```
java \
```


```
  -Xmx216g \
```


```
  -jar rdp_classifier_2.13/rdp_classifier_2.13/dist/classifier.jar \
```


```
  train \
```


```
  -o leray/COInr_WO_Insecta/rdp/trained/ \
```


```
  -s leray/COInr_WO_Insecta/rdp/COInr_WO_Insecta_rdp_trainseq.fasta \
```


```
  -t leray/COInr_WO_Insecta/rdp/COInr_WO_Insecta_rdp_taxon.txt
```
```

#### COInr-Med

```
```
xxxxxxxxxx
```


```
java \
```


```
  -Xmx216g \
```


```
  -jar rdp_classifier_2.13/rdp_classifier_2.13/dist/classifier.jar \
```


```
  train \
```


```
  -o leray/COInr_Med/rdp/trained/ \
```


```
  -s leray/COInr_Med/rdp/COInr_Med_rdp_trainseq.fasta \
```


```
  -t leray/COInr_Med/rdp/COInr_Med_rdp_taxon.txt
```
```

#### COInr-Med+

```
```
xxxxxxxxxx
```


```
java \
```


```
  -Xmx216g \
```


```
  -jar rdp_classifier_2.13/rdp_classifier_2.13/dist/classifier.jar \
```


```
  train \
```


```
  -o leray/COInr_Med_plus/rdp/trained/ \
```


```
  -s leray/COInr_Med_plus/rdp/COInr_Med_plus_rdp_trainseq.fasta \
```


```
  -t leray/COInr_Med_plus/rdp/COInr_Med_plus_rdp_taxon.txt
```
```

### 4.2.2 Taxonomic assigment with RDP classifier

Create output directory

```
```
xxxxxxxxxx
```


```
mkdir -p taxassign/rdp
```
```

#### COInr

```
```
xxxxxxxxxx
```


```
java \
```


```
  -Xmx216g \
```


```
  -jar rdp_classifier_2.13/rdp_classifier_2.13/dist/classifier.jar \
```


```
  classify \
```


```
  -t leray/COInr/rdp/trained/rRNAClassifier.properties \
```


```
  -o taxassign/rdp/COInr_rdp_taxassign.tsv \
```


```
  metafiles/Data_S3.fasta
```
```

#### COInr-WO-Insecta

```
```
xxxxxxxxxx
```


```
java \
```


```
  -Xmx216g \
```


```
  -jar rdp_classifier_2.13/rdp_classifier_2.13/dist/classifier.jar \
```


```
  classify \
```


```
  -t leray/COInr_WO_Insecta/rdp/trained/rRNAClassifier.properties \
```


```
  -o taxassign/rdp/COInr_WO_Insecta_rdp_taxassign.tsv \
```


```
  metafiles/Data_S3.fasta
```
```

#### COInr-Med

```
```
xxxxxxxxxx
```


```
java \
```


```
  -Xmx216g \
```


```
  -jar rdp_classifier_2.13/rdp_classifier_2.13/dist/classifier.jar \
```


```
  classify \
```


```
  -t leray/COInr_Med/rdp/trained/rRNAClassifier.properties \
```


```
  -o taxassign/rdp/COInr_Med_rdp_taxassign.tsv \
```


```
  metafiles/Data_S3.fasta
```
```

#### COInr-Med+

```
```
xxxxxxxxxx
```


```
java \
```


```
  -Xmx216g \
```


```
  -jar rdp_classifier_2.13/rdp_classifier_2.13/dist/classifier.jar \
```


```
  classify \
```


```
  -t leray/COInr_Med_plus/rdp/trained/rRNAClassifier.properties \
```


```
  -o taxassign/rdp/COInr_Med_plus_rdp_taxassign.tsv \
```


```
  metafiles/Data_S3.fasta
```
```

## 4.3 Taxonomic assignment with QIIME2

### 4.3.1 Import database sequences and taxonomy to QIIME2

#### COInr

```
​x


```
qiime tools import \
```


```
  --type 'FeatureData[Sequence]' \
```


```
  --input-path leray/COInr/qiime/COInr_qiime_trainseq.fasta \
```


```
  --output-path leray/COInr/qiime/COInr_sequences.qza
```


```

```


```
qiime tools import \
```


```
  --type 'FeatureData[Taxonomy]' \
```


```
  --input-format HeaderlessTSVTaxonomyFormat \
```


```
  --input-path leray/COInr/qiime/COInr_qiime_taxon.txt \
```


```
  --output-path leray/COInr/qiime/COInr_taxonomy.qza
```
```

#### COInr-WO-Insecta

```
```
xxxxxxxxxx
```


```
qiime tools import \
```


```
  --type 'FeatureData[Sequence]' \
```


```
  --input-path leray/COInr_WO_Insecta/qiime/COInr_WO_Insecta_qiime_trainseq.fasta \
```


```
  --output-path leray/COInr_WO_Insecta/qiime/COInr_WO_Insecta_sequences.qza
```


```

```


```
qiime tools import \
```


```
  --type 'FeatureData[Taxonomy]' \
```


```
  --input-format HeaderlessTSVTaxonomyFormat \
```


```
  --input-path leray/COInr_WO_Insecta/qiime/COInr_WO_Insecta_qiime_taxon.txt \
```


```
  --output-path leray/COInr_WO_Insecta/qiime/COInr_WO_Insecta_taxonomy.qza
```
```

#### COInr-Med

```
```
xxxxxxxxxx
```


```
qiime tools import \
```


```
  --type 'FeatureData[Sequence]' \
```


```
  --input-path leray/COInr_Med/qiime/COInr_Med_qiime_trainseq.fasta \
```


```
  --output-path leray/COInr_Med/qiime/COInr_Med_sequences.qza
```


```

```


```
qiime tools import \
```


```
  --type 'FeatureData[Taxonomy]' \
```


```
  --input-format HeaderlessTSVTaxonomyFormat \
```


```
  --input-path leray/COInr_Med/qiime/COInr_Med_qiime_taxon.txt \
```


```
  --output-path leray/COInr_Med/qiime/COInr_Med_taxonomy.qza
```
```

#### COInr-Med+

```
```
xxxxxxxxxx
```


```
qiime tools import \
```


```
  --type 'FeatureData[Sequence]' \
```


```
  --input-path leray/COInr_Med_plus/qiime/COInr_Med_plus_qiime_trainseq.fasta \
```


```
  --output-path leray/COInr_Med_plus/qiime/COInr_Med_plus_sequences.qza
```


```

```


```
qiime tools import \
```


```
  --type 'FeatureData[Taxonomy]' \
```


```
  --input-format HeaderlessTSVTaxonomyFormat \
```


```
  --input-path leray/COInr_Med_plus/qiime/COInr_Med_plus_qiime_taxon.txt \
```


```
  --output-path leray/COInr_Med_plus/qiime/COInr_Med_plus_taxonomy.qza
```
```

### 4.3.2 Import the test ASV dataset to QIIME artifact

Sequences should be in CAPITAL letters

```
```
xxxxxxxxxx
```


```
qiime tools import \
```


```
--type 'FeatureData[Sequence]' \
```


```
--input-path metafiles/Data_S3.fasta \
```


```
--output-path metafiles/Data_S3.qza
```
```

### 4.3.3 Train Qiime classifier

#### COInr

```
```
xxxxxxxxxx
```


```
qiime feature-classifier fit-classifier-naive-bayes \
```


```
  --i-reference-reads leray/COInr/qiime/COInr_sequences.qza \
```


```
  --i-reference-taxonomy leray/COInr/qiime/COInr_taxonomy.qza \
```


```
  --o-classifier leray/COInr/qiime/COInr_trained.qza
```
```

#### COInr-WO-Insecta

```
```
xxxxxxxxxx
```


```
qiime feature-classifier fit-classifier-naive-bayes \
```


```
  --i-reference-reads leray/COInr_WO_Insecta/qiime/COInr_WO_Insecta_sequences.qza \
```


```
  --i-reference-taxonomy leray/COInr_WO_Insecta/qiime/COInr_WO_Insecta_taxonomy.qza \
```


```
  --o-classifier leray/COInr_WO_Insecta/qiime/COInr_WO_Insecta_trained.qza
```
```

#### COInr-Med

```
```
xxxxxxxxxx
```


```
qiime feature-classifier fit-classifier-naive-bayes \
```


```
  --i-reference-reads leray/COInr_Med/qiime/COInr_Med_sequences.qza \
```


```
  --i-reference-taxonomy leray/COInr_Med/qiime/COInr_Med_taxonomy.qza \
```


```
  --o-classifier leray/COInr_Med/qiime/COInr_Med_trained.qza
```
```

#### COInr-Med+

```
```
xxxxxxxxxx
```


```
qiime feature-classifier fit-classifier-naive-bayes \
```


```
  --i-reference-reads leray/COInr_Med_plus/qiime/COInr_Med_plus_sequences.qza \
```


```
  --i-reference-taxonomy leray/COInr_Med_plus/qiime/COInr_Med_plus_taxonomy.qza \
```


```
  --o-classifier leray/COInr_Med_plus/qiime/COInr_Med_plus_trained.qza
```
```

### 4.3.4 Classify (taxassign) with QIIME2 using SKLEARN algorithm

Create output directory

```
```
xxxxxxxxxx
```


```
mkdir -p taxassign/qiime_sklearn
```
```

#### COInr

```
```
xxxxxxxxxx
```


```
qiime feature-classifier classify-sklearn \
```


```
  --i-classifier leray/COInr/qiime/COInr_trained.qza \
```


```
  --i-reads metafiles/Data_S3.qza \
```


```
  --o-classification taxassign/qiime_sklearn/COInr_qiime_sklearn_taxassign.qza
```
```

#### COInr-WO-Insecta

```
```
xxxxxxxxxx
```


```
qiime feature-classifier classify-sklearn \
```


```
  --i-classifier leray/COInr_WO_Insecta/qiime/COInr_WO_Insecta_trained.qza \
```


```
  --i-reads metafiles/Data_S3.qza \
```


```
  --o-classification taxassign/qiime_sklearn/COInr_WO_Insecta_qiime_sklearn_taxassign.qza
```
```

#### COInr-Med

```
```
xxxxxxxxxx
```


```
qiime feature-classifier classify-sklearn \
```


```
  --i-classifier leray/COInr_Med/qiime/COInr_Med_trained.qza \
```


```
  --i-reads metafiles/Data_S3.qza \
```


```
  --o-classification taxassign/qiime_sklearn/COInr_Med_qiime_sklearn_taxassign.qza
```
```

#### COInr-Med+

```
```
xxxxxxxxxx
```


```
qiime feature-classifier classify-sklearn \
```


```
  --i-classifier leray/COInr_Med_plus/qiime/COInr_Med_plus_trained.qza \
```


```
  --i-reads metafiles/Data_S3.qza \
```


```
  --o-classification taxassign/qiime_sklearn/COInr_Med_plus_qiime_sklearn_taxassign.qza
```
```

### 4.3.5 Classify (taxassign) with QIIME2 using BLAST algorithm

Use three different percentage of identity: 0.97, 0.9, 0.8

Create output directory

```
```
xxxxxxxxxx
```


```
mkdir -p taxassign/qiime_blast
```
```

#### COInr

```
```
xxxxxxxxxx
```


```
qiime feature-classifier classify-consensus-blast \
```


```
  --i-metafiles/Data_S3.qza \
```


```
  --i-reference-reads leray/COInr/qiime/COInr_sequences.qza \
```


```
  --i-reference-taxonomy leray/COInr/qiime/COInr_taxonomy.qza \
```


```
  --p-perc-identity 0.97 \
```


```
  --o-classification taxassign/qiime_blast/COInr_qiime_blast_97_taxassign.qza \
```


```
  --verbose
```


```
  
```


```
qiime feature-classifier classify-consensus-blast \
```


```
  --i-metafiles/Data_S3.qza \
```


```
  --i-reference-reads leray/COInr/qiime/COInr_sequences.qza \
```


```
  --i-reference-taxonomy leray/COInr/qiime/COInr_taxonomy.qza \
```


```
  --p-perc-identity 0.90 \
```


```
  --o-classification taxassign/qiime_blast/COInr_qiime_blast_90_taxassign.qza \
```


```
  --verbose
```


```
  
```


```
qiime feature-classifier classify-consensus-blast \
```


```
  --i-metafiles/Data_S3.qza \
```


```
  --i-reference-reads leray/COInr/qiime/COInr_sequences.qza \
```


```
  --i-reference-taxonomy leray/COInr/qiime/COInr_taxonomy.qza \
```


```
  --p-perc-identity 0.80 \
```


```
  --o-classification taxassign/qiime_blast/COInr_qiime_blast_80_taxassign.qza \
```


```
  --verbose
```
```

#### COInr-WO-Insecta

```
```
xxxxxxxxxx
```


```
qiime feature-classifier classify-consensus-blast \
```


```
  --i-metafiles/Data_S3.qza \
```


```
  --i-reference-reads leray/COInr_WO_Insecta/qiime/COInr_WO_Insecta_sequences.qza \
```


```
  --i-reference-taxonomy leray/COInr_WO_Insecta/qiime/COInr_WO_Insecta_taxonomy.qza \
```


```
  --p-perc-identity 0.97 \
```


```
  --o-classification taxassign/qiime_blast/COInr_WO_Insecta_qiime_blast_97_taxassign.qza \
```


```
  --verbose
```


```
  
```


```
qiime feature-classifier classify-consensus-blast \
```


```
  --i-metafiles/Data_S3.qza \
```


```
  --i-reference-reads leray/COInr_WO_Insecta/qiime/COInr_WO_Insecta_sequences.qza \
```


```
  --i-reference-taxonomy leray/COInr_WO_Insecta/qiime/COInr_WO_Insecta_taxonomy.qza \
```


```
  --p-perc-identity 0.90 \
```


```
  --o-classification taxassign/qiime_blast/COInr_WO_Insecta_qiime_blast_90_taxassign.qza \
```


```
  --verbose
```


```
  
```


```
qiime feature-classifier classify-consensus-blast \
```


```
  --i-metafiles/Data_S3.qza \
```


```
  --i-reference-reads leray/COInr_WO_Insecta/qiime/COInr_WO_Insecta_sequences.qza \
```


```
  --i-reference-taxonomy leray/COInr_WO_Insecta/qiime/COInr_WO_Insecta_taxonomy.qza \
```


```
  --p-perc-identity 0.80 \
```


```
  --o-classification taxassign/qiime_blast/COInr_WO_Insecta_qiime_blast_80_taxassign.qza \
```


```
  --verbose
```
```

#### COInr-Med

```
```
xxxxxxxxxx
```


```
qiime feature-classifier classify-consensus-blast \
```


```
  --i-metafiles/Data_S3.qza \
```


```
  --i-reference-reads leray/COInr_Med/qiime/COInr_Med_sequences.qza \
```


```
  --i-reference-taxonomy leray/COInr_Med/qiime/COInr_Med_taxonomy.qza \
```


```
  --p-perc-identity 0.97 \
```


```
  --o-classification taxassign/qiime_blast/COInr_Med_qiime_blast_97_taxassign.qza \
```


```
  --verbose
```


```
  
```


```
qiime feature-classifier classify-consensus-blast \
```


```
  --i-metafiles/Data_S3.qza \
```


```
  --i-reference-reads leray/COInr_Med/qiime/COInr_Med_sequences.qza \
```


```
  --i-reference-taxonomy leray/COInr_Med/qiime/COInr_Med_taxonomy.qza \
```


```
  --p-perc-identity 0.90 \
```


```
  --o-classification taxassign/qiime_blast/COInr_Med_qiime_blast_90_taxassign.qza \
```


```
  --verbose
```


```
  
```


```
qiime feature-classifier classify-consensus-blast \
```


```
  --i-metafiles/Data_S3.qza \
```


```
  --i-reference-reads leray/COInr_Med/qiime/COInr_Med_sequences.qza \
```


```
  --i-reference-taxonomy leray/COInr_Med/qiime/COInr_Med_taxonomy.qza \
```


```
  --p-perc-identity 0.80 \
```


```
  --o-classification taxassign/qiime_blast/COInr_Med_qiime_blast_80_taxassign.qza \
```


```
  --verbose
```
```

#### COInr-Med+

```
```
xxxxxxxxxx
```


```
qiime feature-classifier classify-consensus-blast \
```


```
  --i-metafiles/Data_S3.qza \
```


```
  --i-reference-reads leray/COInr_Med_plus/qiime/COInr_Med_plus_sequences.qza \
```


```
  --i-reference-taxonomy leray/COInr_Med_plus/qiime/COInr_Med_plus_taxonomy.qza \
```


```
  --p-perc-identity 0.97 \
```


```
  --o-classification taxassign/qiime_blast/COInr_Med_plus_qiime_blast_97_taxassign.qza \
```


```
  --verbose
```


```
  
```


```
qiime feature-classifier classify-consensus-blast \
```


```
  --i-metafiles/Data_S3.qza \
```


```
  --i-reference-reads leray/COInr_Med_plus/qiime/COInr_Med_plus_sequences.qza \
```


```
  --i-reference-taxonomy leray/COInr_Med_plus/qiime/COInr_Med_plus_taxonomy.qza \
```


```
  --p-perc-identity 0.90 \
```


```
  --o-classification taxassign/qiime_blast/COInr_Med_plus_qiime_blast_90_taxassign.qza \
```


```
  --verbose
```


```
  
```


```
qiime feature-classifier classify-consensus-blast \
```


```
  --i-metafiles/Data_S3.qza \
```


```
  --i-reference-reads leray/COInr_Med_plus/qiime/COInr_Med_plus_sequences.qza \
```


```
  --i-reference-taxonomy leray/COInr_Med_plus/qiime/COInr_Med_plus_taxonomy.qza \
```


```
  --p-perc-identity 0.80 \
```


```
  --o-classification taxassign/qiime_blast/COInr_Med_plus_qiime_blast_80_taxassign.qza \
```


```
  --verbose
```
```

# 5. References

Bolyen E, et al. 2019. Reproducible, interactive, scalable and extensible microbiome data science using QIIME 2. Nature Biotechnology 37:852–857. DOI: 10.1038/s41587-019-0209-9.Microbiome, 6, 90.

González,A. et al. (2020) VTAM: A robust pipeline for validating metabarcoding data using internal controls. bioRxiv, 2020.11.06.371187.

Meglécz,E. (2022a) COInr a comprehensive, non-redundant COI database from NCBI-nt and BOLD. DOI: 10.5281/zenodo.6555985.

Meglécz,E. (2022b) COInr and mkCOInr: Building and customizing a non-redundant barcoding reference database from BOLD and NCBI using a lightweight pipeline. BioRxiv:2022.05.18.492423.

Meglécz,E. (2022c) meglecz/mkCOInr: mkCOInr-v.0.2.0. DOI: 10.5281/zenodo.6961340

Wang,Q. et al. (2007) Naive Bayesian classifier for rapid assignment of rRNA sequences into the new bacterial taxonomy. Appl. Environ. Microbiol., 73, 5261–5267.
