# Supplementary figures and images for: Be positive: customized reference databases and new, local barcodes balance false taxonomic assignments in metabarcoding studies
Source: PeerJ. 2023 Jan 9;11:e14616. doi: 10.7717/peerj.14616 (PMC9835706; doi:10.7717/peerj.14616)

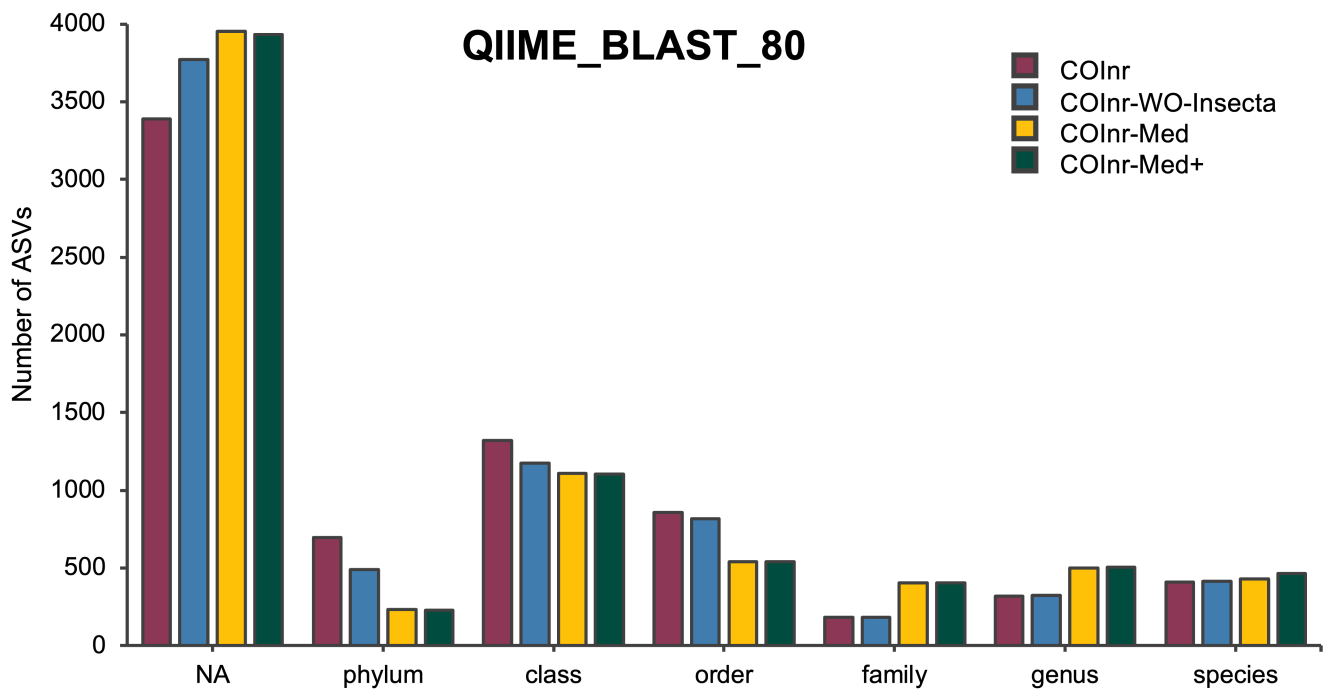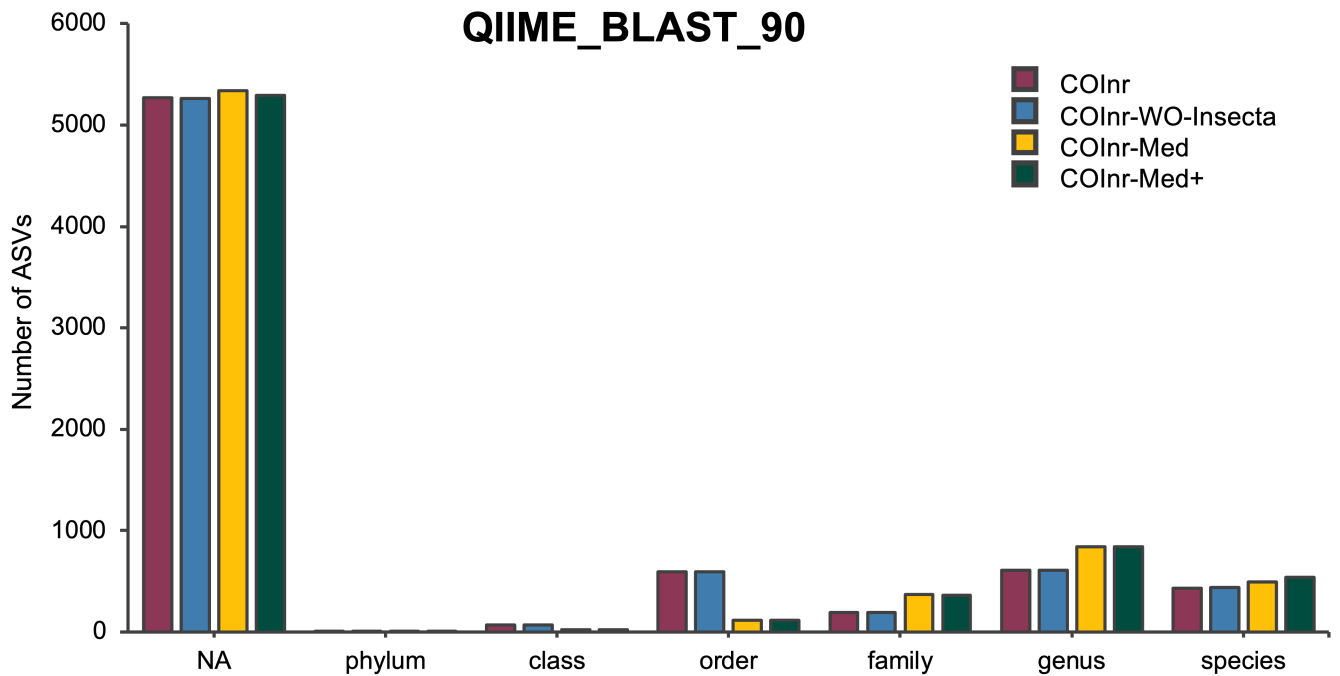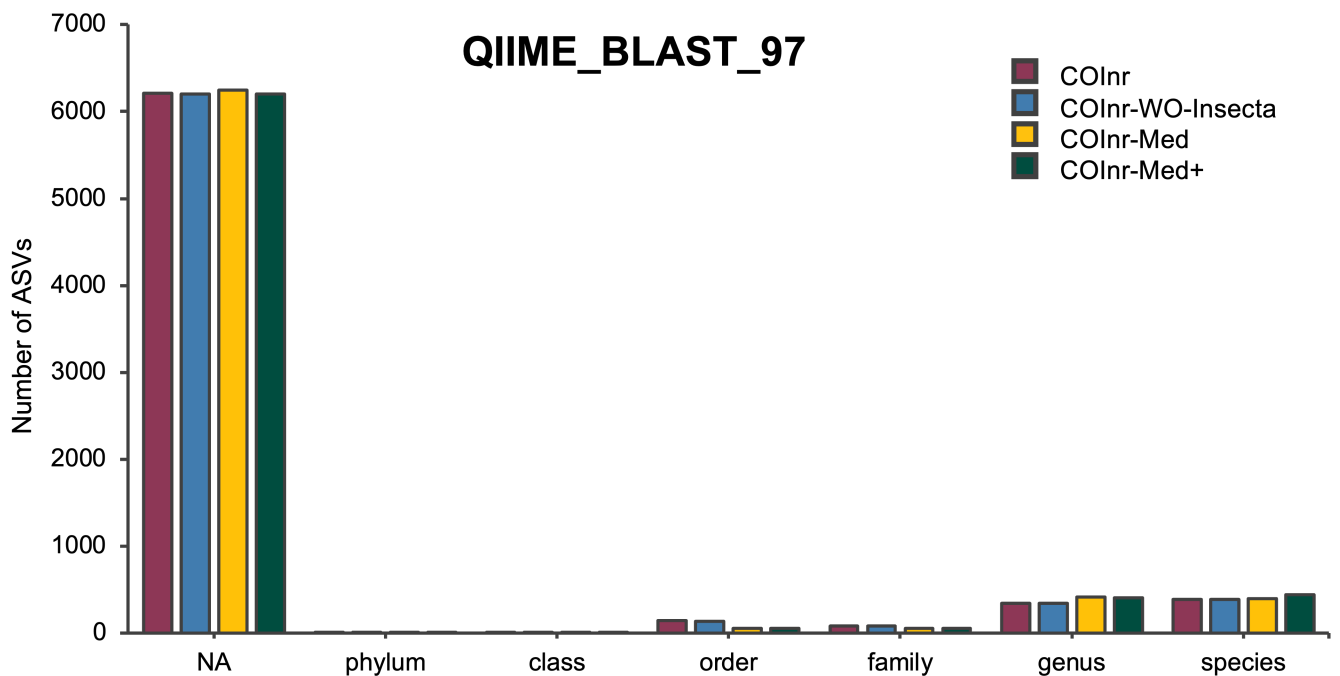

Supplement: Supplemental Information 14 [file peerj-11-14616-s014.pdf]
