# Supplementary material for: Be positive: customized reference databases and new, local barcodes balance false taxonomic assignments in metabarcoding studies
Source: PeerJ. 2023 Jan 9;11:e14616. doi: 10.7717/peerj.14616 (PMC9835706; doi:10.7717/peerj.14616)
Supplement: Supplemental Information 15 — Higher = increase in resolution; Lower = decrease in resolution; NA: unassigned ASVs. Bright colors: compatible changes, Light colors: incompatible changes. [file peerj-11-14616-s015.pdf]

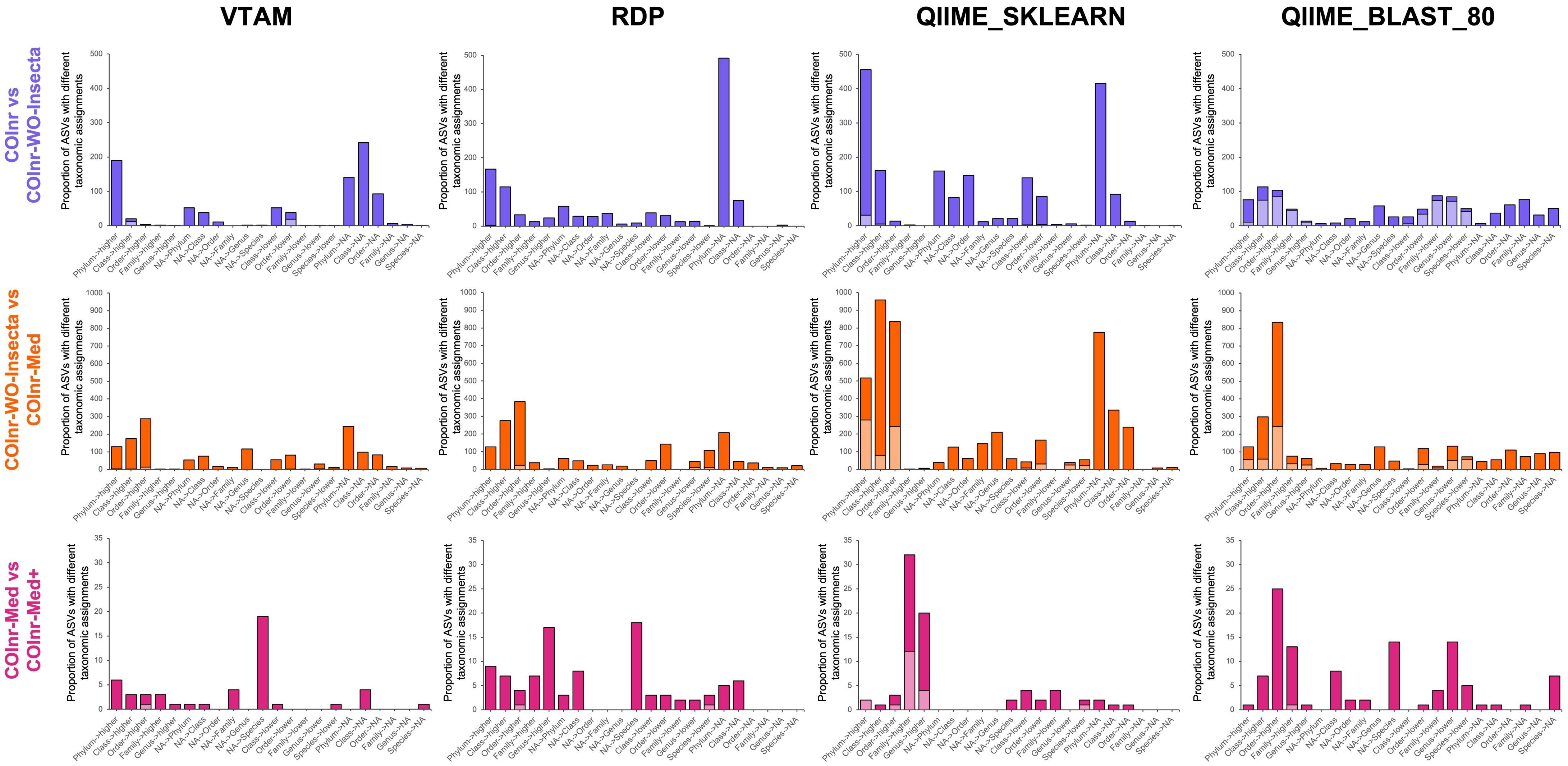
VTAM
RDP
QIIME_SKLEARN
QIIME_BLAST_80
COInr vs COInr-WO-Insecta
COInr-WO-Insecta vs COInr-Med
COInr-Med vs COInr-Med+
Proportion of ASVs with different taxonomic assignments
Phylum->higher
Class->higher
Order->higher
Family->higher
Genus->higher
NA->Phylum
NA->Class
NA->Order
NA->Family
NA->Genus
NA->Species
Class->lower
Order->lower
Family->lower
Genus->lower
Species->lower
Phylum->NA
Class->NA
Order->NA
Family->NA
Genus->NA
Species->NA
